# Streptococcus equi Subspecies zooepidemicus Infective Endocarditis: A Case Report of a Rare Bacterium and Review of the Literature

**DOI:** 10.7759/cureus.98613

**Published:** 2025-12-07

**Authors:** Sara Jochumsen, Julie KK Vishram-Nielsen, Bettina B Pump, Marie B Seibæk, Niels E Bruun

**Affiliations:** 1 Department of Cardiology, Zealand University Hospital, Roskilde, DNK

**Keywords:** antiobiotics, bacteremia, endocarditis, mechanic mitral valve, streptococcus equinus

## Abstract

Infective endocarditis (IE) is a dreaded disease with a high mortality rate. In Denmark, IE is most often caused by *Staphylococcus aureus* or *Streptococcus* species. We present a rare case of a 50-year-old woman with a history of a dual-chamber pacemaker due to third-degree atrioventricular block and a mechanical mitral valve inserted due to mitral stenosis. The patient was admitted to the hospital after she was found lying on the floor in her home. The patient was diagnosed with *Streptococcus equi*subspecies* zooepidemicus *(*S. zooepidemicus*) prosthetic heart valve IE, which was complicated with meningitis, myocardial infarction, and an increasing vegetation load despite antibiotic treatment. The patient underwent heart valve surgery with insertion of a biological mitral valve, and *S. zooepidemicus* was identified in the excised valve tissue. *S*. *zooepidemicus* is a rare zoonotic pathogen in humans. The bacterium is most often found on the skin and mucous membranes of horses and may cause opportunistic infections in horses and other animals. Our literature review found 11 studies reporting 13 cases with *S. zooepidemicus* IE in humans.

## Introduction

Infective endocarditis (IE) is a severe disease, with a largely unchanged mortality rate within the last two decades of 15-30% in-hospital and a mortality rate of 30-40% during the first year after diagnosis [1]. The symptoms are highly variable. Some cases develop acutely with a picture of severe illness with sepsis, rapidly progressive heart failure and unstable hemodynamics due to valve destruction. However, most cases have an insidious course that develops from weeks to months. These patients have diverse and diffuse complaints with low fever, weight loss, musculoskeletal pain, varying degrees of dyspnea, headache, neurological symptoms, and fatigue. Such symptoms are also found in several other chronic conditions, and it is not uncommon for IE patients to have undergone an evaluation for chronic inflammatory disorders or cancer, just as they may have received one or more courses of antibiotics for suspected respiratory or other infections. In addition, approximately 10-20% of IE patients have signs of embolization at the time of diagnosis [1]. In Denmark, Streptococci are implicated in a little more than 30% of IE cases [2]. Streptococci are a heterogeneous group of bacteria with more than 50 different species [3], and certain species have been documented to be more strongly associated with IE than others [3,4]. Previous studies have identified *Streptococcus mitis*/*oralis* and *Streptococcus gallolyticus* (belonging to the Bovis group) among the streptococcal species with the highest IE prevalence and the highest associated IE risk after adjusting for known IE risk factors [4,5]. *S. zooepidemicus* is classified as a gram-positive, β-hemolytic streptococcus, belonging to Lancefield group C. *S. zooepidemicus* is a normal commensal of the oral cavity, pharynx, and respiratory tract of horses and may cause respiratory diseases as an opportunistic pathogen [6]. In a few cases, the bacterium has been isolated in humans as a zoonotic pathogen, although infections are very rare [7]. But severe human infections, including sepsis, meningitis, septic arthritis, or IE, have been described [7].

Here, we present a complicated case of IE with *S. zooepidemicus* in a Danish female patient with repetitive episodes of fever, as well as a literature review of previously described human cases of *S. zooepidemicus* IE.

## Case presentation

A 50-year-old Caucasian woman with a medical history of a mechanical mitral valve due to mitral stenosis, dual-chamber pacemaker due to third-degree atrioventricular block, anxiety, depression, and chronic joint and muscle pain was admitted to the hospital after being found lying with cognitive impairment on the floor at home.

Three days prior to her current hospital admission, the patient had experienced fever, chills, headache, malaise and increasing discomfort due to non-productive cough. The patient had consulted her General Practitioner who suspected that she had the flu. A few months prior to the current hospital admission, the patient had been admitted twice with fever, muscle pain, malaise, and dyspnea and had received treatment for pneumonia with various antibiotic treatments including penicillin. 

Interestingly, the patient owned two horses, which she visited daily for approximately four hours. Additionally, she attended riding lessons at a riding club once a week from August 2024. The club housed around 10-20 horses.

At the time of the current admission, the patient was noticed to have cognitive impairment and after a lumbar puncture she received treatment for meningitis with penicillin and ceftriaxone. The spinal fluid was clear with 332 cells, of which 195 were mononuclear leukocytes and 137 were polynuclear leukocytes. Additionally, lactate in the spinal fluid was 4.00 mmol/L protein 1.40 g/L and *S. zooepidemicus* DNA was identified by 16S PCR. 

**Figure 1:**
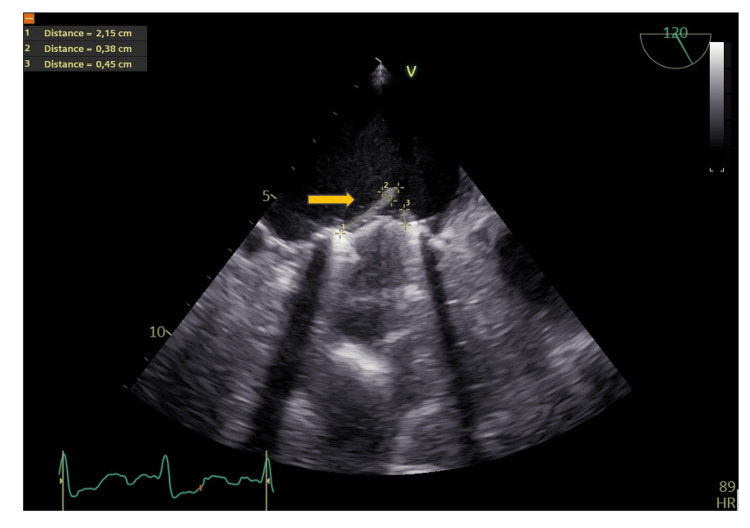
A transesophageal echocardiography (TEE) of the prosthetic mitral valve with visualization of the vegetation three days after hospital admission Arrow shows vegetation with measurements

Blood cultures were drawn at the time of admission and showed three out of three bottles positive for *S. zooepidemicus*. Two bottles were aerobic, and one was anaerobic. Each bottle contained 10 mL of blood. The bacteria were sensitive to penicillin with a minimum inhibitory concentration (MIC) value of 0.032 mg/L and to moxifloxacin, gentamycin, ampicillin and rifampicin with MIC values of 0.250, 32.000, 0.064 and 0.032 mg/L, respectively. Blood cultures were repeated 14 days later and showed no microbial growth. Blood cultures were repeated on three additional instances, and all were without microbial growth. C-reactive protein (CRP) value was increased, whereas leukocytes and thrombocytes were normal, and hemoglobin was low. Furthermore, oxygen saturation and estimated glomerular filtration rate (eGFR) were normal and blood pressure was 104/58 mmHg (see Table 2). There was no audible heart murmur apart from the murmur produced by the mechanical mitral valve. Transesophageal echocardiography (TEE) was performed on suspicion of IE, and showed a 2.15x0.38 cm vegetation on the posterior leaflet of the mitral valve and there was a suspicion of a small vegetation on the anterior leaflet of the mitral valve of 0.45 cm (Figure 1). There were no vegetations on the aortic valve or on the right-sided heart valves. Moreover, the left ventricle was of normal dimension but with reduced ejection fraction of 35%. The patient was known for having a reduced LVEF after the previous mitral valve operation.

**Table 1:** Laboratory and clinical parameters at the time of hospital admission

	Patient’s result	Reference
C-reactive protein	340 mg/L	<8 mg/L
Leukocytes	7.10 x10^9^/L	3.50-8.80x10^9^/L
Thrombocytes	210 x10^9^/L	145-390x10^9^/L
Haemoglobin	6.50 mmol/L	7.30-9.50 mmol/L
Estimated glomerular filtration rate	65 mL/min/1.73 m^2^	>60 mL/min/1.73 m^2^
Saturation	98%	95-100%

**Figure 2:**
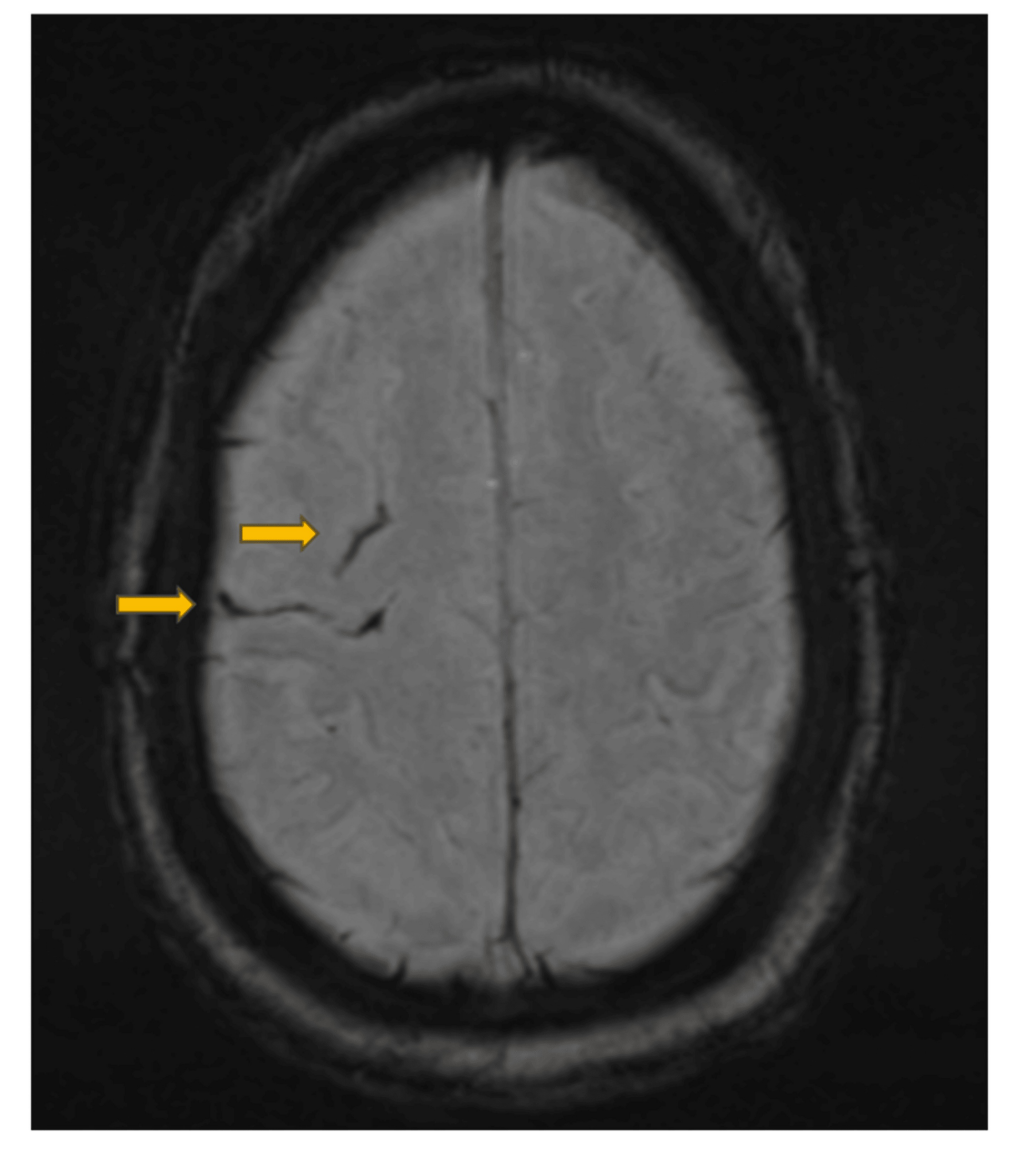
Magnetic resonance imaging (MRI) of the cerebrum Arrows show subarachnoid hemorrhages in two sulci in the right frontal lobe

**Figure 3:**
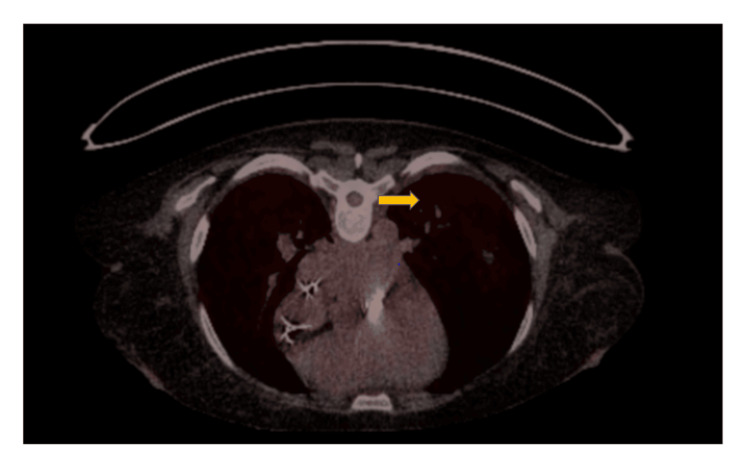
Positron emission tomography/computed tomography (PET/CT) of lungs Arrow indicates pneumonia in left lung

A magnetic resonance imaging (MRI) of the cerebrum demonstrated a minor subarachnoid hemorrhage in the right frontal lobe and a microbleed in the left frontal lobe, but no abscesses or infarcts (Figure 2). On the same day, a positron emission tomography/computed tomography (PET/CT) scan showed positive fluorine-18-fluorodeoxyglucose (F18-FDG) signals in the left lung, indicating pneumonia, and in the esophagus (Figure 3). There was no F-18-FDG activity suggesting infection in the gastrointestinal channel, on the pacemaker generator, or on the electrodes. The patient was referred for further examination of the esophagus, but the F18-FDG signal in the esophagus was considered of no significance.

A dental examination of the patient revealed paradontitis apicalis in one tooth, and the patient was scheduled for extraction of the tooth, which was performed during hospitalization. The tooth was not definitively identified as the cause of IE.

**Figure 4:**
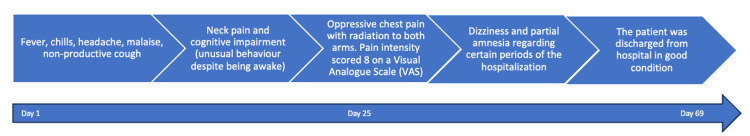
Timeline of clinical symptoms from index admission to end of hospitalization The diagram should be read from left to right.

On Day 25 after admission, the patient experienced central chest pain. An electrocardiogram (ECG) showed paced rhythm, and troponin T (TnT) level was elevated to 41 ng/L. On suspicion of acute coronary syndrome, a coronary angiography was performed, demonstrating an occlusion, most likely an embolus, in the distal part of an obtuse marginal artery, which was treated conservatively. An overview of the patient’s clinical symptoms is presented in Figure 4.

**Figure 5:**
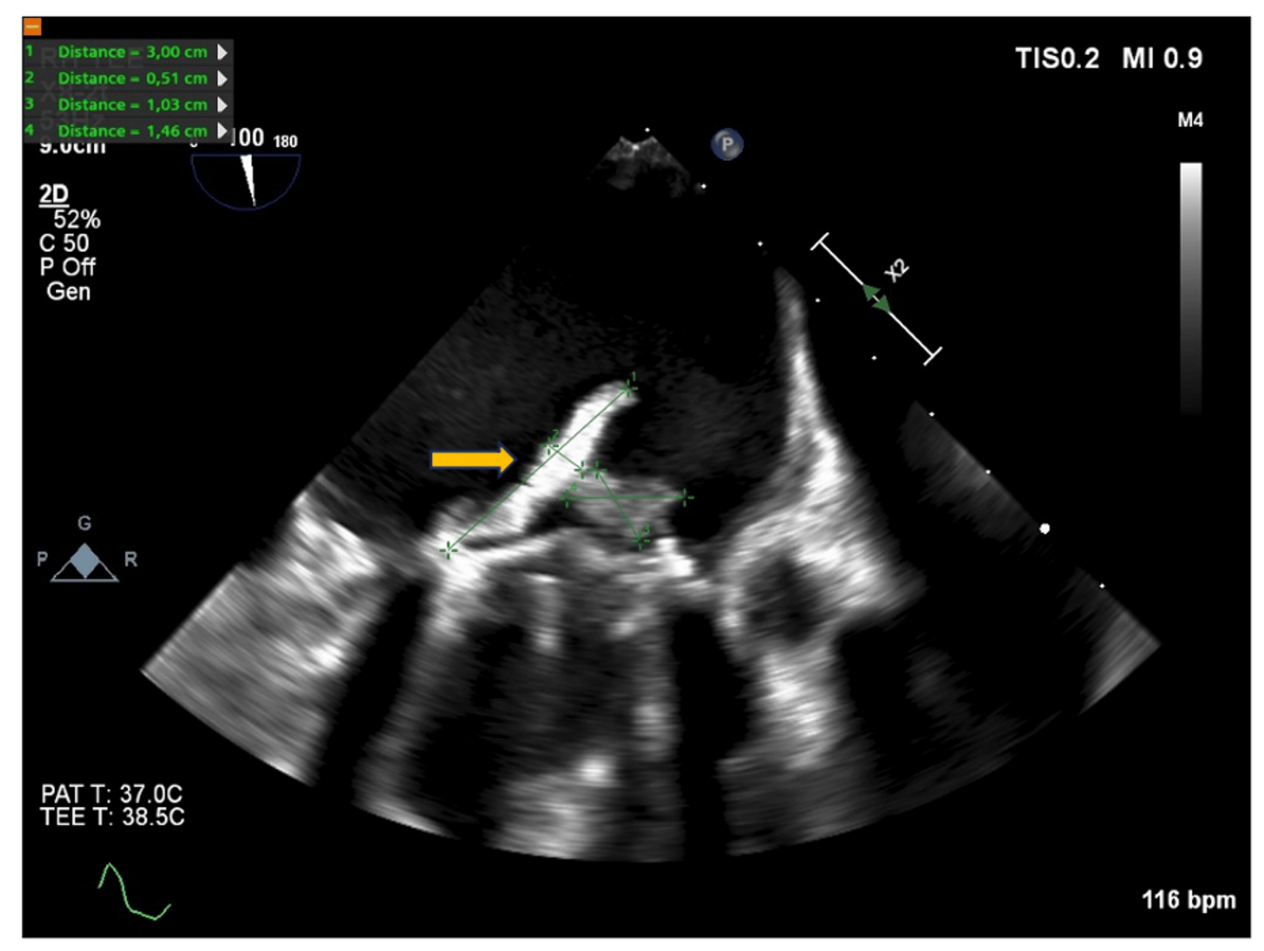
A transesophageal echocardiography (TEE) of the prosthetic mitral valve with visualization of the progression 23 day after hospital admission and prior to surgery Arrow shows vegetation with measurements

Despite continuous treatment with penicillin and moxifloxacin, CRP stagnated around 150 mg/L, and the patient experienced worsening of fever from 38.1 °C to 39.6 °C. High CRP levels during treatment may indicate progression of IE, although it can also represent a reaction to antibiotics, other inflammatory stimuli or infection with additional pathogens. Thorough clinical evaluation and often repeat TEE is required to assess possible complications, including intracardiac progression. Consequently, a new TEE was performed, which showed an increasing vegetation load (Figure 5). The patient was referred to the department of Cardiothoracic Surgery, where she underwent heart valve surgery involving the replacement of the mechanical mitral valve with a biological valve. Cultures from the mitral valve tissue were negative, but *S. zooepidemicus* DNA was identified by 16S polymerase chain reaction (PCR). The dual-chamber pacemaker was removed and replaced with a temporary transvenous pace lead. There was no growth of bacteria from the pacemaker leads. The post-surgical period was without further complications. 

**Figure 6:**
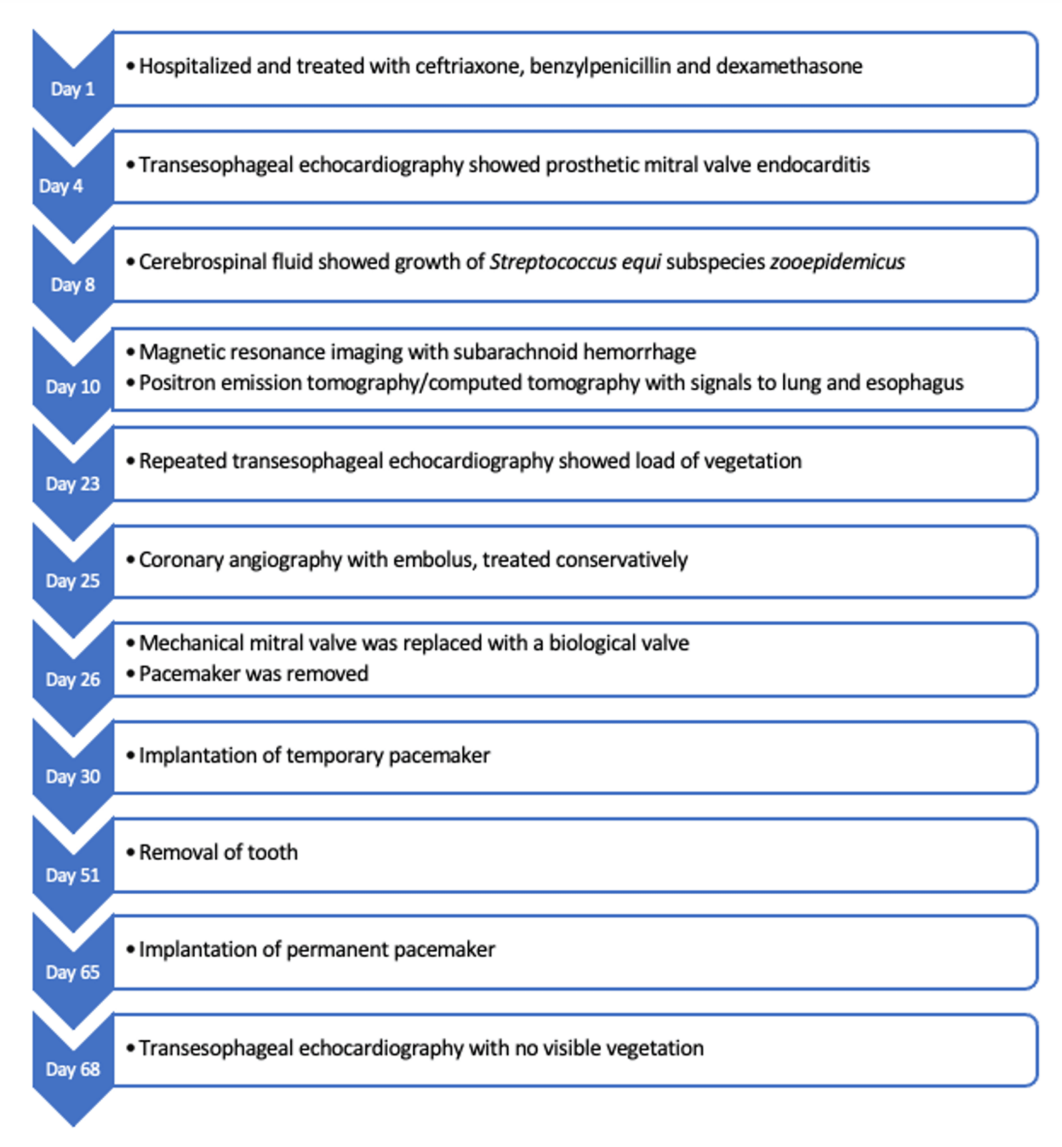
Timeline of investigations, diagnosis, and treatments

The ejection fraction remained around 35% and the temporary pacemaker showed a 100% pace rhythm with a base rate of 50 beats per minute due to third-degree AV node block. Therefore, a biventricular pacemaker was implanted. A TEE was conducted shortly before the patient was discharged from the hospital. The bioprosthetic valve was functioning well with no paravalvular leaks (see Figure 6 for timeline).

## Discussion

Herein, we discuss a Danish female patient with IE caused by *S. zooepidemicus*, a rare bacterium in humans. The patient had several comorbidities and was predisposed to IE due to the presence of a mechanical mitral valve and a dual-chamber pacemaker. She was admitted to the hospital after being found lying confused on the floor at home. Three out of three blood culture bottles were positive for *S. zooepidemicus*, and the patient was diagnosed with definite IE with two major Duke-International Society for Cardiovascular Infectious Diseases (ISCVD) IE criteria: (1) imaging major criteria with a TEE showing a large vegetation on the posterior leaflet of the mitral valve, and (2) surgical major criteria with evidence of IE by direct inspection during the heart valve surgery [8]. Since *S. zooepidemicus *rarely causes IE and was not isolated in repeated blood cultures, a major microbiological criterion was not fulfilled. However, the IE diagnosis was further supported after surgical removal of the mechanical mitral valve, which showed *S. zooepidemicus* DNA by 16S PCR. The patient was treated with penicillin, to which the bacteria were sensitive, with a low minimum inhibitory concentration (MIC) value. After the heart valve surgery, moxifloxacin was prescribed at the discretion of the microbiologists.

*S. zooepidemicus *was isolated for the first time in 1933 by P. R. Edwards, and the bacterium was named animal pyogens A [9]. The United States witnessed the first documented outbreak of *S. zooepidemicus* in humans in 1983. A total of patients had *S. zooepidemicus* isolated from the bloodstream and presented with symptoms such as fever and chills. The source of infection was identified as cheese made from raw milk by cows who had mastitis due to *S. zooepidemicus* [10].

Streptococci can be classified into eight main groups [3]. *Streptococcus equi *belongs to the group of “Other Streptococci” and can be further divided into the following three subspecies: *Streptococcus equi *subspecies *equi*, *Streptococcus equi *subspecies *zooepidemicus,* and *Streptococcus equi *subspecies *ruminatorum* [11]. *S. zooepidemicus’ *genome shares over 92% similarity with the genome of *Streptococcus equi* [6].

Human infections with *S. zooepidemicus *are very rare; however, the colonization of this bacterium in humans mostly leads to severe infections. A study from Finland in 2013 reported three unrelated cases of *S. zooepidemicus *infections among three men, all of whom were working closely with horses. Sepsis occurred in two out of the three men, with one of them also developing meningitis and endocarditis, while the third person experienced purulent arthritis and a psoas abscess. All cases required prolonged antibiotic treatment, surgery, and rehabilitation [7]. These findings are in line with our Danish patient, who had ongoing and close contact with horses and developed a severe infection with several complications, which required prolonged treatment. Both contact with animals and consumption of dairy products have been proposed as entry pathways in previous studies of *S. zooepidemicus*. Five of these studies suspect close contact with horses [7, 11-14], which is consistent with our patient’s weekly interactions with horses. Nine of the 13 cases included in this report survived [7, 11-18], four died [19-20], and three had to undergo surgical treatment of the implicated valve [7, 11, 14]. This aligns with an overall in-hospital mortality rate of up to about 30%, and a rate for heart valve surgery of about 20-25% in IE patients.

With a MIC value of 0.032mg/l, *S. zooepidemicus* in our present case was very sensitive to penicillin. According to guidelines, IE on native valves with streptococci with low MIC values can be treated with penicillin in monotherapy [1]. However, due to the severity of the infection, the microbiologists decided to treat the patient with a combination of two antibiotics.

Our patient was diagnosed with pneumonia, meningitis, and IE. These three conditions together constitute a triad that resembles the Austrian syndrome [21]. Austrian syndrome was first described in 1862, prior to the identification of pathogens [22]. The syndrome was later linked to *Streptococcus pneumonia* in 1975 by Robert Austrian [23]. It has been suggested that the pathophysiology of the syndrome involves septic microemboli, and it is reported that pneumonia often is the first manifestation [21], which aligns with our Danish patient.

A potential underlying mechanism could be the same as indicated by the Austrian syndrome [21], which is that *S. zooepidemicus *colonizes the heart valve by hematogenous spread from the lung. In our case, the patient had prolonged symptoms with coughing and had been treated on the suspicion of a respiratory infection by the general practitioner prior to hospitalization. Furthermore, a PET/CT scan showed F-18-FDG signals in the lungs. A primary focus in the lung could therefore be consistent with hematogenous spread from pneumonia; however, the transmission remains uncertain. A previous study, which investigated 88 cases of Group C streptococci bacteremia, found no specific entry of the bacteria in 34 of the cases but suggests the gastrointestinal tract to be the source of entry in 16 patients, whereas 10 were directly linked to ingestion of contaminated milk, which supports the view of multiple possible entry pathways [24].

Herein, we also present a review of 11 previously described studies of 13 cases of *S. zooepidemicus* IE (Table 2).

**Table 2:** Literature review of infective endocarditis with Streptococcus equi subspecies zooepidemicus Text appears as written in the original article

Year of Publication	Author	Study	Country	Sex and Age	Suspected Source of Contamination	Comorbidities	Symptoms	Implicated Valve	Treatment
2024	Franceschi et al. [11]	Review and case report	Italy	Male, 62 years	Horse	Hypertension and hypercholesterolemia	Intermittent high fever, cough, memory deficits, and confusion	Aortic valve	Aortic valve replacement with a biological prosthesis and intravenous cefotaxime and gentamicin
2018	Høyer- Nielsen et al. [12]	Case report	Faroe Islands	Male, 82 years	Horse	Ischemic heart disease, atrial fibrillation, low malignant prostate cancer, gout, type 2 diabetes, and removal of all teeth in the upper mouth six months prior	Dyspnea, hemoptysis, impaired general condition, and shoulder pain	Aortic valve	Intravenous cefuroxime and benzylpenicillin, oral amoxicillin and rifampicin
2015	Villamil et al. [13]	Case report	Spain	Male, 73 years	Horse	Hypertension, dyslipidemia, diverticulosis, metallic aortic valve prosthesis, dilatation of the ascending aorta with a valved tube	Fever, poor general condition	Aortic valve prosthesis	Intravenous penicillin and gentamicin
2013	Pelkonen et al. [7]	Case report	Finland	Male, 57 years	Horse	Aortic valve insufficiency	Unconscious, febrile	Bicuspid aortic valve	Resection of the aortic valve and intravenous high-dose penicillin and gentamicin
2009	Poulin et al. [14]	Case report	Canada	Female, 59 years	Horse	Hypertension, type 2 diabetes, dyslipidemia, myocardial infarction, chronic renal failure, obesity, left ophthalmic vein thrombosis, atrial septal defect, hypothyroidism, primary hyperparathyroidism	Generalized weakness, lightheadedness when standing up, fever, more dyspneic, vomiting, resting tremors, clear rhinorrhea, cough	Posterior leaflet of the mitral valve	Valve replacement and intravenous ceftriaxone and rifampin
2006	Bordes-Benítez et al. [15]	Clinical and epidemiological study	Spain	Female, 70 years	Inadequately pasteurized cheese	None	Joint pain, gastrointestinal symptoms	Unknown	Beta-lactam agent
2004	Lee et al. [16]	Case report	England	Male, 79 years	Unknown	None	Right-leg pain, fever, severe headache, deteriorating conscious state	Aortic valve	Intravenous penicillin
1990	Yuen et al. [18]	Case report	China	Female, 58 years	Unknown	Unknown	Unknown	Mitral valve	Intravenous benzylpenicillin
1988	Edwards et al. [19]	Case reports	England	Male, 73 and 79 years, female, 52 years	Unpasteurized cow milk	Unknown	Fever, confusion, flu-like illness	Unknown	Cephalosporins, ampicillin, metronidazole, penicillin
1982	Martinez-Luengas et al. [17]	Case report	Spain	Male, 51 years	Animals	Rheumatic heart disease with mitral and aortic valve involvement, occasional pulmonary edema	Fever, malaise	Mitral valve	Intravenous penicillin and intramuscular streptomycin
1980	Ghoneim et al. [20]	Case report	England	Male, 81 years	Animals	Chronic rheumatic heart disease	Retrosternal chest pain, lethargy, malaise, fever, mildly confused	Unknown	Intravenous penicillin, gentamicin, and oral amoxycillin

## Conclusions

Many different bacteria can cause IE, and Streptococci are often implicated. This case demonstrates that if blood cultures show a zoonotic bacterium, the bacterium’s taxonomic group, followed by determination of the species and the relationship with IE in humans, should be considered to help assess the IE risk. In the present instance, *S. zooepidemicus* is a hemolytic streptococcus from Lancefield group C known to be associated with horses, but transmission to humans, causing severe infections, including IE, may seldom occur. Therefore, in case of growth of *S. zooepidemicus* in blood cultures, increased awareness of IE is recommended. In such cases, early echocardiography with extended antibiotic therapy should be considered, especially if there are risk factors of IE such as a prosthetic heart valve or a cardiac implantable electronic device.
